# Childhood BMI trajectories and hypertension in adulthood: a population-based cohort study

**DOI:** 10.1038/s41371-026-01219-5

**Published:** 2026-09-04

**Authors:** Dominique Searle, Elisabeth Wreford Andersen, Julie Aarestrup, Jennifer L. Baker

**Affiliations:** Center for Clinical Research and Prevention, Copenhagen University Hospital - Bispebjerg and Frederiksberg, Frederiksberg, Denmark

**Keywords:** Risk factors, Hypertension

## Abstract

Higher body mass index (BMI) in childhood is associated with hypertension (HTN) especially in young adulthood. We examined whether longitudinal patterns of BMI development in childhood are associated with HTN across adult ages. We included 124 670 children (51% boys), born between 1960–1996, from the Copenhagen School Health Records Register with height and weight measurements at ages 6–15 years. Latent class trajectory models were used to identify five sex-specific BMI trajectories: below-average, average, above-average, overweight, and obesity. Individuals were followed from age 25 in national health registers from 1995–2022 for HTN. Cox models were used to estimate sex-specific hazard ratios (HRs) with 95% confidence intervals (CIs) for HTN across childhood BMI trajectories, with adjustment for birth cohort and parental education. Follow-up was split into ages 25–39, 40–49, and 50–62 years. Over a median 18-year follow-up, 5 970 men (9.4%) and 5 712 women (9.3%) developed HTN. Men and women with above-average, overweight, and obesity childhood BMI trajectories had higher hazards of HTN than those with the average trajectory. Associations attenuated with age but remained significant. Compared to men with the average trajectory, the obesity trajectory was associated with a HR = 4.31 (95% CI: 3.45–5.37) at ages 25–39 and HR = 2.12 (1.63–2.76) at ages 50–62 years. Compared to women with the average trajectory, the obesity trajectory was associated with a HR = 3.53 (2.89–4.30) at ages 25–39 and HR = 2.19 (1.70–2.82) at ages 50–62 years. Childhood BMI trajectories are positively associated with adult HTN in a dose−response manner highlighting the potential value of early obesity prevention.

## Introduction

Hypertension (HTN) is one of the primary modifiable risk factors for cardiovascular disease (CVD), which has been the leading cause of mortality globally for over three decades [1]. While obesity in adulthood is a well-established contributor to CVD-related morbidity, including HTN [2], the long-term effects of obesity in childhood remain less well understood. Understanding how childhood body size influences adult HTN is essential for the earlier identification, prevention, and treatment of obesity and its related comorbidities. The steep rise in overweight and obesity rates in children over the last years, outpacing that of adults, further highlights its importance as a public health priority [3].

As the most commonly used surrogate measure of adiposity, body mass index (BMI) in childhood has been widely linked to long-term diseases, including type 2 diabetes, coronary heart disease, and certain cancer forms [4, 5]. Several, but not all, studies have also linked a higher BMI in childhood to an elevated risk of HTN in adulthood [6–9]. However, these findings on this association remain inconclusive due to methodological differences and drawbacks, such as short follow-up durations into adulthood, heterogeneity in the timing of BMI and HTN assessment, and reliance on a single BMI value early in life, which fails to capture changes in body size during this critical period of development [6, 7]. A life-course approach that captures cumulative exposure can help overcome some of these limitations. Particularly, latent class trajectory modelling (LCTM) is useful to identify classes in populations that follow different trajectories over time, such as BMI patterns [10]. Few studies have examined the relationship between childhood BMI trajectories and adult HTN [11–14], as most have focused on HTN outcomes in adolescence [15–17], often limited by small sample sizes and attrition bias due to large follow-up losses.

As childhood obesity continues to increase across all world regions [3], there is a need to assess developmental patterns of adiposity and subsequent HTN in large, population-based cohorts. Using routinely collected health data, we examined whether distinct BMI trajectories across childhood are associated with the risk of developing HTN in adulthood in an unselected cohort of schoolchildren after a median follow-up of 18 years. Furthermore, we examined whether parental education levels influenced this relationship.

## Materials and methods

### Cohort

This cohort study used data from the Copenhagen School Health Records Register (CSHRR), which includes information on schoolchildren born between 1930 and 1996 who ever attended public or private schools in Copenhagen, Denmark [18]. Since the 1930s, children in Copenhagen have received regular school health examinations from school entry (ages 6–7 years) through school exit (ages 13–15 years). During these examinations, trained school physicians or nurses weighed children without shoes and either naked or in light clothing (to the nearest 100 g) and measured heights (to the nearest 0.5 cm). Originally, the health examinations were annual, and their frequency decreased over time to three times including at school entry and exit. Since April 2, 1968, every resident in Denmark upon birth or immigration has been assigned a government-issued personal identification number by the Danish Civil Registration System (CRS) [19]. This number was used to link individuals to national registers to obtain information on HTN and parental education.

### Exposure: childhood BMI trajectories

The main exposure in this study was sex-specific childhood BMI trajectories, which have been previously developed for the CSHRR using LCTMs [20] (Figure S1). These trajectories are sex-specific to account for growth differences between boys and girls. From 2 and up to 12 BMI values per child were used (≥ 95% had more than 2) from ages 6–15 years. Trajectories were modelled using natural splines, with knots at ages 8, 10, and 12 years, corresponding approximately to the 25th, 50th, and 75th percentiles of the age distribution of the children. This approach was chosen to best capture the natural growth patterns of children at these ages and to evenly distribute the knot points across the age range. The model was adjusted for 5-year birth cohorts to account for potential variations in BMI development related to birth year. The optimal number of BMI trajectories was determined based on several fit indices and parameters including the Bayesian Information Criterion, relative entropy, mean posterior probability, and visual inspection of mean BMI trajectory plots. In the final model, which did not include random effects, five non-crossing BMI trajectories were identified for boys and girls and are referred to as: below-average, average, above-average, overweight, and obesity.

### Outcome

The Danish healthcare system provides free, tax-funded services, including primary, secondary and hospital care [21]. To assess the outcome of a HTN diagnosis, we linked each individual’s personal identification number to the Danish National Patient Register (DNPR) and the Danish National Prescription Registry (NPR). The DNPR, established in 1977, includes hospital discharge diagnoses coded according to the International Classification of Diseases, 10th revision (ICD-10) since 1994 [22]. The NPR, established in 1994, includes data on prescriptions filled at Danish pharmacies, using Anatomical Therapeutic Chemical Classification System (ATC) codes to register each medication [23]. In Denmark, general practitioners serve as the main contact point for patients, and are therefore the primary prescribers of antihypertensive medications.

HTN was defined as either: 1) the date of the first hospital ICD-10 diagnosis in the DNPR, or 2) the filling of at least one prescription for a minimum of 2 different antihypertensive drug classes within a 1-year period, as recorded in the NPR. The prescription-based criterion has previously been validated, with a reported positive predictive value of 88%, a sensitivity of 75%, and a specificity of 96% for HTN [22]. We used the following ICD-10 codes to define hospital-diagnosed HTN: DI10, DI11, DI12, DI13, DI14, DI15. Antihypertensive specific medications were defined as all within the classes of angiotensin-converting enzyme inhibitors (C09A, C09B), angiotensin II receptor blockers (C09C, C09D), beta-blockers (C07), calcium channel blockers (C07FB, C08, C09BB, C09DB), and thiazides (C03A, C03EA, C07B, C07D), excluding medications specifically to treat heart failure, namely trandolapril (C09AA10, C09BB10), valsartan/sacubitril (C09DX04), and nebivolol (C07AB12, C07BB12, C07FB12, C09DX05).

### Covariates

We constructed a directed acyclic graph (DAG) using the software DAGitty to represent the assumed relationships between childhood BMI trajectories and HTN (Figure S2). Based on these a priori assumptions, we identified potential confounders and mediators. As presented in the DAG, the minimal sufficient adjustment sets for estimating the total effect of childhood BMI trajectories on adult HTN includes birth cohort and parental education (as a proxy for the child’s socioeconomic environment).

Parental education was defined as the highest educational attainment of either parent, which was obtained from the Danish Educational Attainment Register. We categorized highest parental education as follows: low (less than primary, primary, and lower secondary education), medium (upper secondary and post-secondary education), and high (tertiary education). In addition, we included birth year (in 5-year cohorts) as a covariate to account for potential differences in BMI development related to birth year.

### Follow-up and study population

**Fig. 1:**
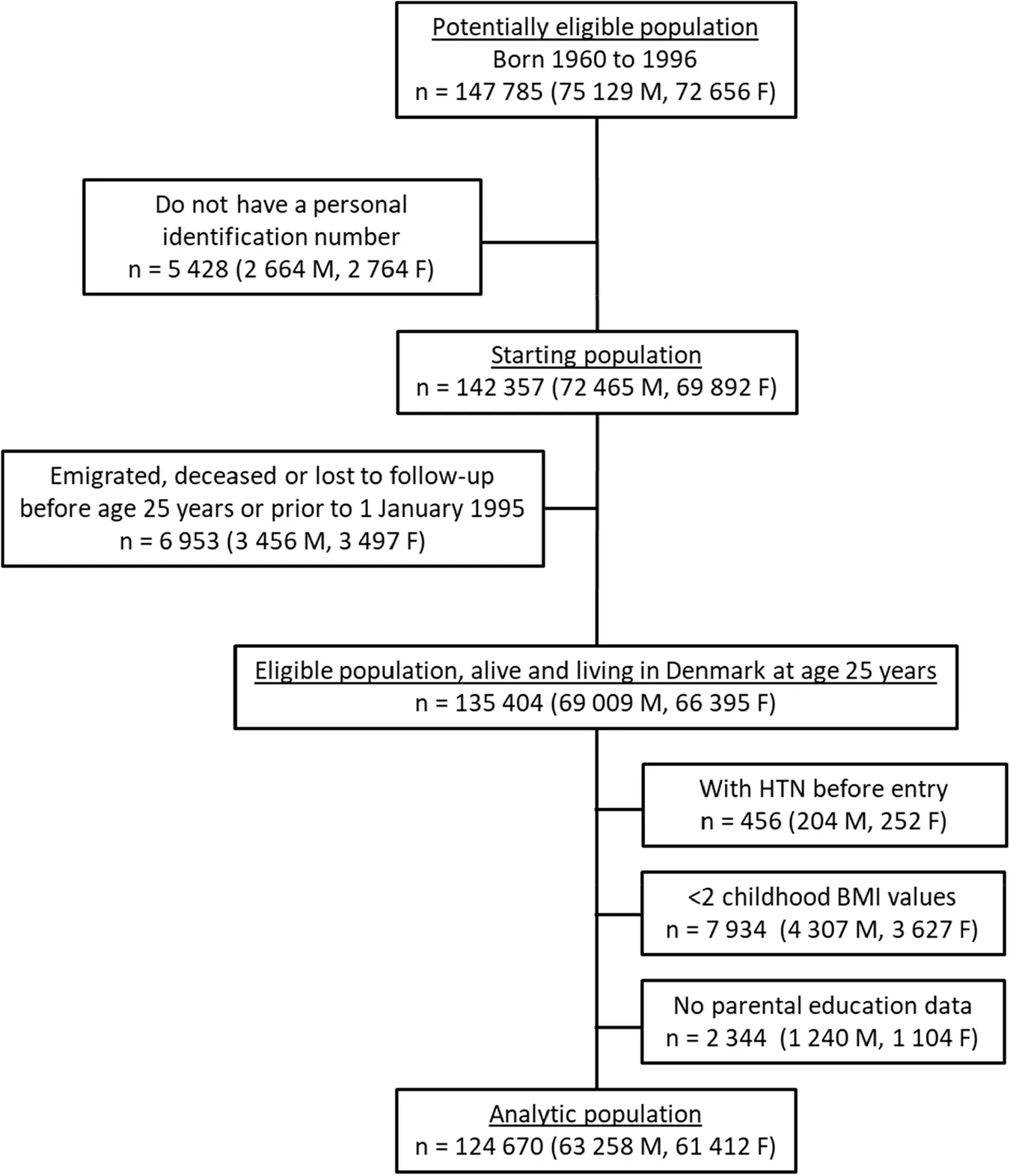
Flow diagram of the individuals from the Copenhagen School Health Records Register (CSHRR) included in the study. M: male; F: female; HTN: hypertension; BMI: body mass index.

We included individuals born from 1960–1996 in our study as reliable coverage of information on parental education was not available before this time. From January 1, 1995, data in the NPR are considered complete and valid [23]. Therefore, we followed individuals from this date or their 25th birthday, whichever happened last, until the first occurrence of a HTN diagnosis, HTN by prescription, death, emigration, loss to follow-up, or June 30, 2022 (latest available register data), whichever occurred first. To be eligible for this study, individuals were required to be born between 1960 and 1996 (*n* = 147 785) and have a valid personal identification number (*n* = 142 357). Individuals who were excluded included those who had HTN prior to the start of follow-up (*n* = 456), those who died (*n* = 1 095), emigrated (*n* = 5 848), or were lost to follow-up (*n* = 10) before study entry. We excluded individuals with prior HTN as HTN at these ages is more likely to reflect secondary, congenital, or non-obesity-related aetiologies. The final analytic study population consists of *n* = 124 670 individuals (61 412 women, 49.3%) (Fig. 1).

### Statistical analyses

The baseline characteristics are summarized using medians and interquartile ranges (IQRs) for continuous variables and counts with percentages for categorical variables. These descriptive statistics are presented by sex and by childhood BMI trajectory groups based on the highest posterior probability (modal assignment).

We used Cox proportional hazard regression models to examine the associations between childhood BMI trajectories and HTN in adulthood. To account for potential misclassification, the posterior probabilities assigned to each trajectory (rather than modal assignment) were used as exposures. The results are expressed as hazard ratios (HRs) with 95% confidence intervals (CIs), relative to the average trajectory group. We present two different models. The initial model was adjusted for 5-year birth cohorts. The adjusted model additionally included the highest educational attainment of either parent.

We assessed the proportional hazards assumption underlying the Cox model using graphical checks of cumulative Martingale residuals. In both sexes, the assumption was not met for the overweight and obesity trajectories, as the effect of the BMI trajectories on HTN decreased with adult age. To address this violation, we split follow-up time into two (25–39 and ≥40 years) and three (25–39, 40–49, and 50–62 years) age bands, respectively, and compared their fit using likelihood ratio tests. The three-age-band model provided the best fit for both men and women and all analyses were conducted using this age split. All analyses were conducted in R (version 4.4.1).

## Results

A total of 124 670 individuals (49.3% women) with BMI values in childhood (ages 6–15 years) were included in the study. Most men were classified in the average BMI trajectory (37.4%), followed by the above-average (24.1%), below-average (22.8%), overweight (11.4%), and obesity (4.2%) BMI trajectories (Table 1). The distribution was similar for women: average (35.5%), above-average (24.9%), below-average (22.0%), overweight (12.7%), and obesity (4.8%). Medium parental education was the most common across all BMI trajectories. However, a socioeconomic gradient was observed across BMI trajectory groups. Individuals with lower parental education were more likely to belong to higher BMI trajectory groups, while those with higher parental education were predominantly in lower BMI trajectory groups (Table 1).

**Table 1:** Characteristics of individuals (*n* = 124 670) in the CSHRR by childhood BMI trajectory and sex*.

		Childhood BMI trajectory				
Characteristic	Total	Below-average	Average	Above-average	Overweight	Obesity
**Women**						
*n* (%)	61 412 (49.3)	13 525 (22.0)	21 828 (35.5)	15 311 (24.9)	7 782 (12.7)	2 966 (4.8)
Birth year, median (IQR)	1977 (1967–1988)	1977 (1967–1989)	1975 (1967–1987)	1976 (1967– 1987)	1979 (1968–1989)	1985 (1974–1991)
Age at HTN, years, median (IQR)	46.5 (40.2–51.8)	47.0 (41.0–52.3)	47.4 (41.1–52.5)	46.8 (40.6–51.8)	45.1 (38.4–50.5)	42.6 (34.8–47.7)
Highest parental education, *n* (%)						
Low education	14 581 (23.7)	3 057 (22.6)	5 074 (23.2)	3 561 (23.3)	2 036 (26.2)	853 (28.8)
Medium education	24 974 (40.7)	5 287 (39.1)	8 704 (39.9)	6 386 (41.7)	3 247 (41.7)	1 350 (45.5)
High education	21 857 (35.6)	5 181 (38.3)	8 050 (36.9)	5 364 (35.0)	2 499 (32.1)	763 (25.7)
Hypertension, *n* (%)	5 712 (9.3)	1 027 (7.6)	1 867 (8.6)	1 547 (10.1)	908 (11.7)	363 (12.2)
**Men**						
*n* (%)	63 258 (50.7)	14 442 (22.8)	23 687 (37.4)	15 269 (24.1)	7 231 (11.4)	2 629 (4.2)
Birth year, median (IQR)	1977 (1967–1988)	1977 (1967–1989)	1975 (1967–1987)	1976 (1967–1988)	1982 (1971–1990)	1986 (1977–1992)
Age at HTN, years, median (IQR)	47.6 (41.8–53.0)	48.5 (42.7–53.8)	48.0 (42.7–53.6)	48.0 (42.3–53.1)	45.3 (39.1–51.3)	42.3 (36.6–48.2)
Highest parental education, *n* (%)						
Low education	14 977 (23.7)	3 311 (22.9)	5 527 (23.3)	3 651 (23.9)	1 773 (24.5)	715 (27.2)
Medium education	25 507 (40.3)	5 723 (39.6)	9 392 (39.7)	6 218 (40.7)	3 015 (41.7)	1 159 (44.1)
High education	22 774 (36.0)	5 408 (37.4)	8 768 (37.0)	5 400 (35.4)	2 443 (33.8)	755 (28.7)
Hypertension, *n* (%)	5 970 (9.4)	1 120 (7.8)	2 199 (9.3)	1 572 (10.3)	776 (10.7)	303 (11.5)

Data are presented as medians and interquartile ranges (IQRs) or as numbers with corresponding percentages.

*CSHRR* Copenhagen School Health Records Register; *BMI* body mass index, *HTN* hypertension.

*To characterize individuals within each BMI trajectory, individuals were assigned to the trajectory for which they had the highest posterior probability, based on modal assignment.

**Fig. 2:**
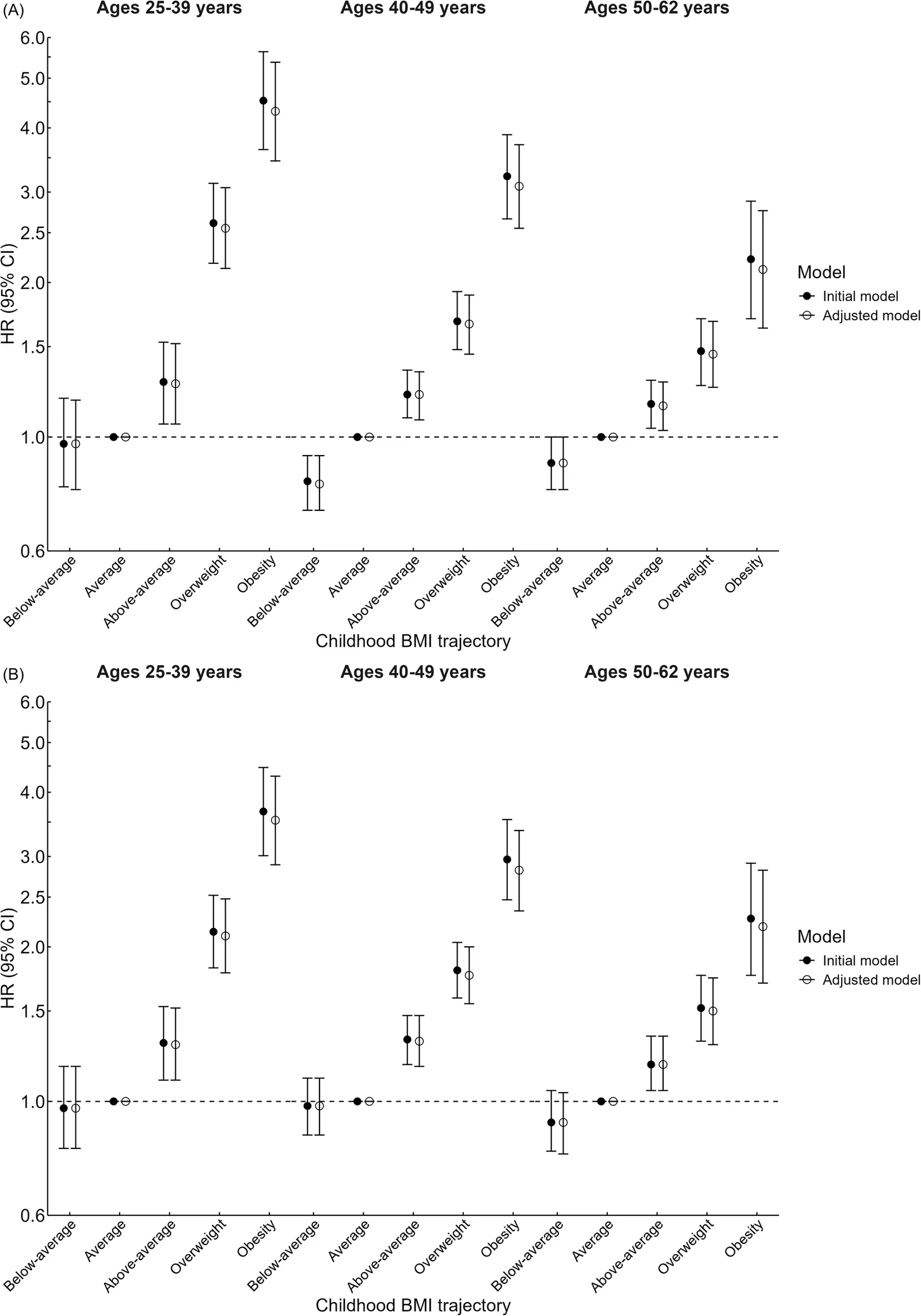
Forest plots of hazard ratios (HRs) for the association between childhood body mass index (BMI) trajectories and adult hypertension by age group. The HRs with their corresponding 95% confidence intervals (CIs) were estimated using Cox proportional hazards regression models and expressed relative to the average childhood BMI trajectory, among men (**A**) and women (**B**). The initial model was adjusted for 5-year birth cohorts and the adjusted model adittionally included the highest attained parental education by either parent.

During a median follow-up of 18 years (25^th^–75th percentiles: 12–23 years), 11 682 individuals developed HTN between ages 25 and 62 years, including 5 970 (9.4%) men and 5 712 (9.3%) women. The median age at HTN diagnosis was 48 years (IQR: 42–53) for men and 47 years (IQR: 40–52) for women. In both sexes, there was a dose−response relationship between childhood BMI trajectories and the hazard of HTN in adulthood (Fig. 2 and Table 2). Individuals with the above-average, overweight and obesity trajectories during childhood had an increased hazard of HTN across all age groups (25–39, 40–49, and 50–62 years) compared with the average childhood BMI trajectory. The associations were strongest in the youngest adults and attenuating but remaining significant in older age groups. Adjustment for parental education did not materially change the associations. In men, the childhood obesity trajectory was associated with the highest hazard of HTN, particularly at ages 25–39 years (adjusted HR = 4.31, 95% CI: 3.45–5.37) compared to the average BMI trajectory. The hazard declined with age but remained elevated at ages 50–62 years (adjusted HR = 2.12, 95% CI: 1.63–2.76). Men with the above-average and overweight BMI trajectories in childhood showed similar age-related HTN risk patterns. Men with the below-average BMI trajectory had a significantly reduced HTN hazard in the 40–49 and 50–62 age groups, but not at 25–39 years (Table 2). Among women, the highest HTN hazard was also observed at ages 25–39 years with the childhood obesity trajectory (adjusted HR = 3.53, 95% CI: 2.89–4.30), with lower hazards at older adult ages. No significant association was found for the below-average BMI trajectory in women at any ages.

**Table 2:** Associations between BMI trajectories in childhood and hypertension in adulthood by sex and age group*.

			Initial model ^†^	Adjusted model ^‡^
Sex	Childhood BMI trajectory	Ages (years)	HR (95% CI)	HR (95% CI)
Women	Below-average	25–39	0.97 (0.81−1.17)	0.97 (0.81–1.17)
		40–49	0.98 (0.86–1.11)	0.98 (0.86–1.11)
		50–62	0.91 (0.80–1.05)	0.91 (0.79–1.04)
	Average		1 [Reference]	1 [Reference]
	Above-average	25–39	1.30 (1.10–1.53)	1.29 (1.10–1.52)
		40–49	1.32 (1.18–1.47)	1.31 (1.17–1.47)
		50–62	1.18 (1.05–1.34)	1.18 (1.05–1.34)
	Overweight	25–39	2.14 (1.82–2.52)	2.10 (1.78–2.48)
		40–49	1.80 (1.59–2.04)	1.76 (1.55–2.00)
		50–62	1.52 (1.31–1.76)	1.50 (1.29–1.74)
	Obesity	25–39	3.67 (3.01–4.47)	3.53 (2.89–4.30)
		40–49	2.96 (2.47–3.54)	2.82 (2.35–3.37)
		50v62	2.27 (1.76–2.91)	2.19 (1.70–2.82)
Men	Below-average	25–39	0.97 (0.80–1.19)	0.97 (0.79–1.18)
		40–49	0.82 (0.72–0.92)	0.81 (0.72–0.92)
		50–62	0.89 (0.79–1.00)	0.89 (0.79–1.00)
	Average		1 [Reference]	1 [Reference]
	Above-average	25–39	1.28 (1.06–1.53)	1.27 (1.06–1.52)
		40–49	1.21 (1.09–1.35)	1.21 (1.08–1.34)
		50–62	1.16 (1.04–1.29)	1.15 (1.03–1.28)
	Overweight	25–39	2.61 (2.18–3.12)	2.55 (2.13–3.06)
		40–49	1.68 (1.48–1.92)	1.66 (1.45–1.89)
		50–62	1.47 (1.26–1.70)	1.45 (1.25–1.68)
	Obesity	25–39	4.52 (3.63–5.63)	4.31 (3.45–5.37)
		40–49	3.22 (2.66–3.88)	3.08 (2.55–3.71)
		50–62	2.22 (1.70–2.88)	2.12 (1.63–2.76)

*BMI* body mass index, *HR* hazard ratio, *CI* confidence interval.

*The associations were estimated using Cox proportional hazards models.

^†^The initial model was adjusted for birth cohort (5-year intervals).

^‡^The adjusted model was adjusted for birth cohort and highest parental education (low, medium, or high).

## Discussion

In this large population-based study, we found a dose−response relationship between childhood BMI trajectories at ages 6–15 years and the hazard of developing HTN in adulthood for both men and women. Compared to the average childhood BMI trajectory, the above-average, overweight, or obesity BMI trajectories were associated with a significantly increased hazard of HTN, and this was particularly pronounced at younger adult ages. Conversely, the below-average BMI trajectory was associated with a reduced hazard of HTN among men aged 40–62 years, but not among women. If the current trends in childhood overweight and obesity continue rising as projected [3], these results suggest that the burden of adult HTN may substantially increase, especially at younger ages.

Our results support the hypothesis that having increased adiposity across childhood is associated with a higher risk of developing HTN in adulthood. Direct comparisons with previous studies are, however, challenging as few studies align with our approach of examining BMI trajectories within childhood and HTN outcomes across a wide range of ages in adulthood [12, 14]. Among those with childhood information, most studies evaluated outcomes during adolescence or early adulthood [14–16, 24, 25], which does not capture the incidence of HTN later in life when the risk may be greater. These studies analysed 3–7 distinct crossing BMI trajectory groups, including both stable and ascending and descending patterns. Ascending BMI trajectories are generally associated with increased risks of elevated blood pressure in adolescence compared with stable trajectories. In contrast, other BMI trajectory studies did not find any associations [13, 26], which may be due to limited statistical power. In our cohort, we identified 5 non-crossing BMI trajectories, meaning each child stayed roughly in the same BMI percentile over time relative to their peers. Overall, our findings align with those from several cohort studies which examined BMI at single or multiple ages [6, 7] and with trajectory analyses from child to early and mid-life [8, 27].

We found a novel age-dependent effect, whereby the hazard of HTN in individuals with above-average BMI trajectories was particularly high at younger adult ages and diminished but remained significantly elevated at older adult ages. This attenuation might be explained by a longer time gap between the exposure and the outcome, and the influence of additional competing risk factors with age. Moreover, the stronger age-dependent association observed among the youngest suggests that a higher adiposity in childhood has a particularly strong influence in the development of early-onset HTN, which is already identifiable in young adulthood. This is additionally supported by recent reports showing that primary (i.e., essential) HTN related to obesity, instead of secondary, has become the leading form of paediatric HTN [28]. The earlier onset of HTN is particularly concerning, as it implies a greater lifetime exposure to elevated blood pressure and a higher cumulative burden.

It is likely that the associations we identified may be modified by subsequent patterns of BMI development across adult life. However, information on adult BMI was not available for the entire cohort, and we therefore could not evaluate the entire BMI trajectory from childhood into adulthood. Although changes in body size during adulthood may partly mediate the association between childhood BMI and adult HTN, our findings are unlikely to be explained solely by tracking of body size across the life course. Supporting this interpretation, a previous study using a subsample (*n* = 98 211) of the same cohort found that BMI tracked only moderately from childhood into young adulthood and weakly into later adulthood, whereas most adults with obesity did not have obesity as children [29]. Similarly, a landmark study by Juonala et al. pooled data from four cohorts with BMI assessed in childhood and adulthood and found that adults with obesity who had overweight or obesity in childhood had a higher risk of HTN than those with obesity with a normal childhood BMI (relative risk, 2.7 [95% CI: 2.2–3.3] vs. 2.1 [1.7–2.4]), suggesting that childhood adiposity has a residual effect on HTN risk. Nonetheless, individuals with high childhood BMI who did not have obesity in adulthood had a similar HTN risk to those who had consistently normal BMI [30]. Consistent with these findings, studies on BMI trajectories across the life course (e.g., ages 6–50 years) generally show a more than twofold higher risk of HTN in adulthood for individuals following normal-to-overweight or persistently overweight trajectories compared to those with stable normal weight trajectories, whereas this risk appears reversed in children with overweight who achieve a normal BMI in adulthood [27]. Nonetheless, due to the considerable heterogeneity in the number of BMI values, their temporal distribution across the life course, their timing relative to the HTN assessment and the modelling approaches used in these studies, the critical age at which BMI gain or loss impacts HTN is unknown.

As with most chronic diseases, current guidelines recommend lifestyle modification as the cornerstone of HTN prevention and treatment, focusing on body weight control, a healthy diet, avoidance of smoking and alcohol, regular exercise, sufficient sleep, and stress management [31]. The most recent *Lancet* Commission on HTN highlighted that meaningful and sustained reductions in the burden of elevated blood pressure cannot be achieved through clinical treatment and adult lifestyle interventions alone, but instead require a life course approach to prevention, beginning as early as conception [32]. Our findings support the importance of the life course approach, highlighting childhood and adolescence as key periods for HTN prevention and management, particularly among individuals with overweight and obesity.

The mechanisms underlying obesity-induced HTN at adult ages remain under investigation, but several key pathways have been identified. These include the activation of the sympathetic nervous system (the fight-or-flight response) due to impaired baroreceptor sensitivity, elevated free fatty acids and hormonal influences (e.g., insulin and leptin); changes in kidney function, including impaired pressure natriuresis and the renin-angiotensin-aldosterone system activation; and hormonal impairment and chronic inflammation (i.e., insulin, leptin, adiponectin, among others), all contributing to sodium retention, vascular dysfunction, and endothelial dysfunction [33]. As autopsy findings from landmark studies like the Bogalusa Heart Study have shown that early atherosclerotic changes—including fatty streaks and fibrous plaques in the aorta and coronary arteries—are associated with elevated BMI and other cardiovascular risk factors in children and young adults [34], it is plausible that the early exposure to high BMI in childhood has a mechanistic link with HTN.

Several factors, including socioeconomic and lifestyle influences, may also be related to high BMI and elevated blood pressure. In our study we adjusted for the highest parental education as a proxy for the child’s socioeconomic environment in childhood. The estimates changed very little between the initial and adjusted models, which may be partly due to the relatively small differences in parental education across BMI trajectory groups. Denmark’s homogenous population and welfare system may mitigate the effects of socioeconomic disparities on health outcomes.

A key strength of our study is the use of a large, population-based cohort, which is representative of school-aged children. Furthermore, our study leverages data from Denmark’s universal and free healthcare system, which reduces the risk of selection bias related to healthcare access. Through time, Denmark has maintained nationwide registers by the routine collection of administrative and health data of high quality [21]. Therefore, we were able to link each individual’s personal identifier across these registers, allowing a complete capture of HTN cases and censoring due to death or lost-to follow up, thereby minimizing attrition and information bias. Moreover, the inclusion of prescription and hospital registry-based HTN diagnoses, rather than self-reported outcomes, further increases clinical validity. We used a systematic life-course analysis to examine longitudinal BMI values in a well-characterized cohort of Copenhagen schoolchildren. This approach enabled the identification of five distinct BMI trajectories, highlighting subgroups at greater subsequent risk of HTN.

Limitations of the present study include the use of BMI. Although BMI might not be a reliable indicator of fat mass at the individual level, it is regarded as a useful proxy for adiposity at the population level [35]. In addition to BMI, we lacked data on other anthropometric factors such as waist circumference or waist-to-height ratio (WHtR). It is worth mentioning that while WHtR has been proposed as a superior indicator of cardiometabolic risk in adults, studies in children and adolescents show that BMI is slightly stronger associated with HTN and blood pressure or performs similarly to WHtR with other risk factors [36]. Another consideration is that HTN was identified from hospital diagnoses and prescription records rather than directly from blood pressure measurements. Thus, we were unable to identify individuals with elevated blood pressure who had not received a clinical diagnosis or been prescribed an antihypertensive medication, which may have resulted in some under-ascertainment of HTN. However, such misclassification would most likely be non-differential with respect to the childhood BMI trajectories and would generally tend to attenuate the observed associations. Nevertheless, the direction and magnitude of any resulting bias cannot be determined with certainty.

We cannot exclude that residual confounding remains, as in all observational studies. Although a mediation analysis with adult risk factors (e.g., high BMI, smoking, physical inactivity) would be of interest, this was not the aim of our study, and these variables were unavailable. However, while adjusting for adult BMI and other factors is common in obesity research, doing so may obscure the true effect of childhood BMI as risk factors in adulthood occur *after* childhood body size and therefore lie on the causal pathway to later disease development. Controlling for variables along this causal path can introduce overadjustment bias [37]. Lastly, the generalizability of these findings is limited to other white populations.

In this study, we showed that above-average BMI trajectories in childhood are associated with increased risks of HTN in adulthood, and notably at younger ages. Although the hazard of HTN decreased with advancing age, it remained significantly elevated across all adult age groups. Furthermore, only among men, a below-average BMI trajectory showed a reduced hazard of HTN.

Our findings support the developmental origins of HTN, indicating that the underlying processes are likely to begin early in life. The dose−response relationship observed across all the above-average trajectories and adult age groups suggests that even a BMI below the levels defined as overweight by international contemporary criteria may be associated with elevated risks of HTN later in life. We recommend that future research examines the mediating role of adult risk factors and assess the generalizability in more ethnically diverse populations. Given that childhood BMI trajectories are early-life markers of HTN risk, and HTN is the strongest modifiable risk factor for CVD, focusing on prevention and treatment efforts for overweight and obesity in early life represents a plausible and potentially effective strategy to help reduce the future burden of HTN and CVD.

## Summary

### What is known about the topic

- Hypertension (HTN) is a major modifiable risk factor for cardiovascular disease. Obesity in adulthood is a well-established risk factor for CVD-related morbidity, including HTN.
- A high BMI in childhood has also been associated with several adverse outcomes in adulthood. However, the results on its relationship with adult HTN remain inconsistent.
- Most previous studies had limited follow-up into adulthood and relied on a single BMI value in childhood, not accounting for the dynamic changes in body size across childhood.
- A life course approach that captures cumulative exposure can help overcome these limitations.

### What this study adds

- Among the largest population-based studies to link distinct trajectories of BMI across childhood to HTN in adulthood over long-term follow-up with few missing individuals.
- In men and women, HTN risk is elevated even below conventional childhood overweight and obesity thresholds.
- The association between childhood BMI trajectories and HTN is strongest in younger adulthood.

## Supplementary information

Supplemental material

## Data Availability

The data underlying this article cannot be shared publicly due to the privacy of individuals included in the study. The data may be available on reasonable request and pending approval from the steering committee at the Center for Clinical Research and Prevention, Copenhagen University Hospital – Bispebjerg and Frederiksberg, Capital Region, Denmark, which governs the use of this data.

## Code availability

The code used for the analyses is not publicly available but is available from the corresponding author upon reasonable request.
