# Supplementary material for: Childhood BMI trajectories and hypertension in adulthood: a population-based cohort study
Source: J Hum Hypertens. 2026 Sep 4;40(11):895–902. doi: 10.1038/s41371-026-01219-5 (PMC13747808; doi:10.1038/s41371-026-01219-5)
Supplement: Supplementary file 1 [file 41371_2026_1219_MOESM1_ESM.docx]

**Supplemental Figures**

**Figure S1.** Estimated mean childhood body mass index trajectories among children born 1950-1954. (A) Girls. (B) Boys.

**A. Girls**

**B. Boys**

Figure adapted from: Aarestrup J, Blond K, Vistisen D, Jørgensen ME, Frimodt-Møller M, Jensen BW, et al. (2022) Childhood body mass index trajectories and associations with adult-onset chronic kidney disease in Denmark: A population-based cohort study. PLoS Med 19(9): e1004098. https://doi.org/10.1371/journal.pmed.1004098, licensed under CC BY 4.0.

**Figure S2.** Directed acyclic graph (DAG) representing the association between body mass index (BMI) trajectories across childhood, key confounding and mediating variables, and adult hypertension. The pink arrows represent the biasing paths while causal paths are indicated by green arrows. Ancestors of an outcome are drawn in blue and ancestors of both exposure and outcomes are drawn in red. The minimal sufficient adjustment sets for estimating the total effect of childhood BMI trajectories on adult hypertension includes birth cohort and parental education.
